# Advances in antibody-mediated rejection of renal allografts

**DOI:** 10.3389/fimmu.2026.1951717

**Published:** 2026-09-18

**Authors:** Bin Song, Junjie Sun, Chao Hu, Xuyang Liu

**Affiliations:** 1 Linyi Health School of Shandong Province, Linyi, China; 2 Department of Andrology, Linyi People’s Hospital, Shandong, China; 3 Department of Renal Transplantation, Linyi People’s Hospital, Shandong, China; 4 Institute of Transplantation Medicine, The Second Affiliated Hospital of Guangxi Medical University, Guangxi Clinical Research Center for Organ Transplantation, Guangxi Key Laboratory of Organ Donation and Transplantation, Nanning, China

**Keywords:** antibody-mediated rejection, complement system, donor-specific antibodies, endothelial cell injury, kidney transplantation

## Abstract

Antibody-mediated rejection (AMR) remains a major obstacle to long-term kidney allograft survival. It does not arise from a single pathway. Preformed or *de novo* donor-specific antibodies (DSA), endothelial injury, microvascular inflammation, complement-dependent and complement-independent mechanisms, and chronic remodeling may each contribute, often in different combinations. Recent Banff updates, biopsy-based transcriptomics, DSA assessment, and donor-derived cell-free DNA (dd-cfDNA) testing have made AMR phenotypes easier to recognize. Still, these tests are most useful when interpreted together rather than treated as separate answers. The same caution applies to treatment. Plasma exchange, intravenous immunoglobulin, B-cell or plasma-cell directed agents, complement inhibition, IL-6 pathway blockade, and optimization of maintenance immunosuppression differ in the strength of their evidence, the patients they may help, their toxicities, and their feasibility in routine practice. This review summarizes current concepts in AMR pathogenesis, diagnosis, and emerging therapy, using phenotype-guided assessment to separate expert-consensus practice from randomized evidence, observational experience, and experimental interventions.

## 1 Introduction

Patients with end-stage renal disease show substantial improvement in quality of life and long-term survival after kidney transplantation (1), but long-term allograft survival remains limited by antibody-mediated rejection (AMR), a major cause of graft loss (2). AMR can arise from donor-specific antibodies (DSA) against donor human leukocyte antigens (HLA) or, in some cases, from non-HLA antibody-associated endothelial injury (2, 3). Pre-existing DSA is strongly associated with early graft injury, whereas *de novo* DSA, often linked to underimmunosuppression or nonadherence, is particularly relevant to late active or chronic active AMR (3–5). After binding to graft endothelium, antibodies may trigger classical complement activation, antibody-dependent cellular cytotoxicity, Fc receptor-mediated immune-cell recruitment, and direct endothelial signaling, ultimately leading to microvascular inflammation, transplant glomerulopathy, interstitial fibrosis/tubular atrophy, and progressive graft dysfunction (6, 7). Recent high-throughput approaches, including single-cell sequencing, proteomics, and biopsy-based transcriptomics, have provided new views of the cellular and molecular landscape of AMR (8–10). For example, Lionet et al. used transcriptomic analysis of repeat biopsies obtained before and after extracorporeal photopheresis (ECP), an ex vivo cell-based immunomodulatory procedure, in patients with chronic antibody-mediated kidney rejection and identified changes in fibrotic and inflammatory transcriptomic biomarkers such as CAV1, CD19, and IL-21 (11). As the field moves from empirical treatment toward mechanism- and phenotype-guided care, this review discusses AMR pathogenesis, diagnostic strategies, therapeutic evidence, and future research priorities.

### 1.1 Methods for literature selection

This narrative review was developed to synthesize clinically relevant advances in AMR after kidney transplantation. We searched PubMed, Web of Science, and Google Scholar for English-language publications using combinations of the terms “kidney transplantation”, “antibody-mediated rejection”, “donor-specific antibody”, “Banff”, “microvascular inflammation”, “transcriptomics”, “donor-derived cell-free DNA”, “plasma exchange”, “intravenous immunoglobulin”, “rituximab”, “bortezomib”, “carfilzomib”, “eculizumab”, “C1 esterase inhibitor”, “tocilizumab”, “clazakizumab”, “felzartamab”, “daratumumab”, “belatacept”, and “non-HLA antibodies”. The final search was performed on June 16, 2026.

### 1.2 Phenotype framework of AMR

AMR is better understood as a group of related phenotypes than as a single clinicopathological entity. The term covers clinically different settings: injury associated with preformed or *de novo* DSA, early or late presentation, active or chronic active disease, symptomatic or subclinical disease, HLA or non-HLA antibodies, and complement-dependent or complement-independent mechanisms. These categories often overlap, but they are not interchangeable. They differ in pathogenesis, diagnostic certainty, prognosis, and response to treatment. Early active AMR with preformed DSA is often complement driven, so antibody removal and complement blockade are most relevant in this setting. Late chronic active AMR is usually a different clinical problem. It is often linked to *de novo* DSA, nonadherence, long-lived plasma cells, and established transplant glomerulopathy, and it tends to respond poorly to the same interventions used earlier in the disease course. In this review, we use these phenotypes to structure the discussion and to separate accepted clinical practice from off-label use and experimental approaches.

## 2 Immunological mechanisms of AMR

### 2.1 Generation and regulation of DSA

DSA production is central to AMR. Activation and regulation of B cells and plasma cells occur through coordinated interactions among donor antigen, B-cell receptors, follicular helper T cells (Tfh), germinal-center reactions, IL-21, BAFF, APRIL, and regulatory immune-cell networks (12–16). Recent studies suggest that BAFF signaling reflects the immunologic risk profile after kidney transplantation, and BAFF/APRIL pathways may influence B-cell maturation and plasma-cell survival, although direct therapeutic evidence remains limited (17, 18). Memory B cells may rapidly differentiate into antibody-secreting cells after antigen re-exposure, contributing to active AMR, whereas long-lived plasma cells can maintain persistent DSA production and contribute to late or chronic active AMR (19). These mechanisms provide biologically plausible targets, but clinical evidence for directly modifying these pathways in established kidney allograft AMR remains limited. Targeting the Tfh-B-cell axis, BAFF/APRIL signaling, or immune regulatory pathways should therefore be presented as mechanism-informed and phenotype-dependent rather than universally established treatment.

### 2.2 Role of the complement system in AMR

Complement activation is an important effector mechanism in some AMR phenotypes, particularly when complement-binding DSA and C4d deposition are present (20–23). Upon binding to HLA antigens on graft endothelial cells, some DSA can trigger the classical complement pathway and generate C3a, C5a, and membrane attack complex, promoting endothelial injury and inflammatory-cell recruitment. However, DSA-mediated injury is not uniformly complement dependent (24). Fc gamma receptor-mediated NK-cell activation, macrophage recruitment, endothelial activation, and antibody-induced intracellular signaling can also drive microvascular inflammation and chronic remodeling (24–27) (**Figure 1**). This distinction is clinically relevant because complement inhibition is unlikely to fully control complement-independent injury.

**Figure 1:**
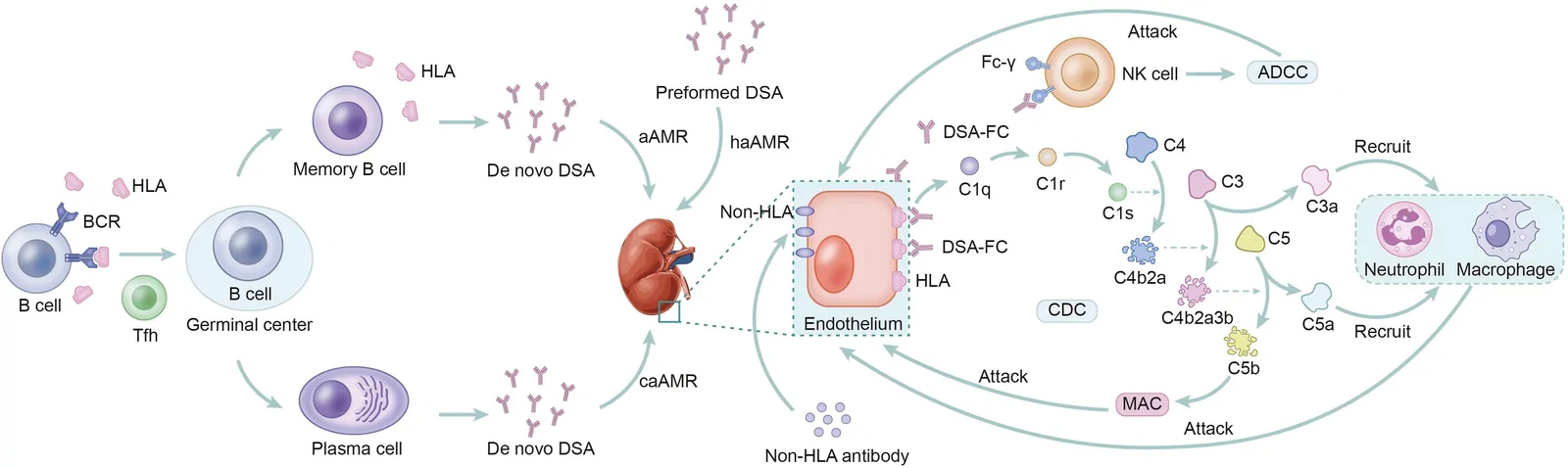
Mechanisms of DSA generation and antibody-mediated endothelial injury in kidney allograft rejection. HLA, human leukocyte antigen; BCR, B-cell receptor; Tfh, T follicular helper cell; DSA, donor-specific antibody; AMR, antibody-mediated rejection; aAMR, active AMR; caAMR, chronic active AMR; haAMR, hyperacute AMR; ADCC, antibody-dependent cellular cytotoxicity; CDC, complement-dependent cytotoxicity; Fc-γ, Fc gamma receptor; MAC, membrane attack complex; NK cell, natural killer cell.

### 2.3 Endothelial cell injury and microvascular remodeling

Following antibody binding or immune-cell engagement, endothelial injury can initiate microvascular inflammation and chronic remodeling (28). Injured endothelial cells and surrounding graft tissue release mediators and extracellular matrix components that may promote peritubular capillary basement membrane multilayering, transplant glomerulopathy, interstitial fibrosis/tubular atrophy, and progressive loss of graft function (10, 29–31). Endothelial-to-mesenchymal transition may contribute to this fibrotic process, although its relative contribution in human AMR requires careful interpretation (25, 32). Early recognition of endothelial injury and chronic remodeling is therefore important, but treatment response should not be inferred from short-term fluctuations in creatinine, DSA MFI, or isolated histologic activity alone.

## 3 Progress in the diagnosis of AMR

### 3.1 Serological DSA monitoring

DSA testing is central to AMR risk assessment, but MFI should not be read as a direct measure of antibody level. In the Luminex single-antigen bead assay, antibody binding is reported as mean fluorescence intensity (MFI), which is a semi-quantitative readout rather than a standardized antibody concentration or titer (33–35). That signal is influenced by assay conditions and by how the antigen is displayed on the bead, including bead composition, antigen density, epitope presentation, reactivity with denatured HLA, complement interference, saturation, serum treatment, prozone effects, and variation between laboratories. Therefore, isolated MFI thresholds or short-term MFI changes should not be used as stand-alone evidence of pathogenicity or treatment response. Interpretation is strongest when DSA class, specificity, persistence, kinetics, complement-binding capacity, epitope or eplet context, graft function, proteinuria, and biopsy findings are considered together. C1q- and C3d-binding assays may enrich risk stratification by identifying DSA associated with complement activation, but they should be regarded as risk-associated tools rather than definitive measures of DSA pathogenicity (36–38).

### 3.2 Histopathological diagnosis

The Banff classification provides an integrated diagnostic framework for AMR rather than a requirement that histological, serological, and molecular evidence must always be simultaneously present (39–41). Contemporary Banff concepts emphasize morphological evidence of microvascular injury, evidence of antibody interaction with the endothelium, circulating DSA when detectable, C4d staining, validated biopsy-based transcript diagnostics where available, and exclusion of alternative causes of endothelial injury. Molecular assessment can support the diagnosis, particularly in diagnostically challenging or C4d-negative cases, but it is not mandatory in every case and should not be considered a stand-alone definitive test. The Banff 2022 report highlighted phenotypes such as probable AMR and microvascular inflammation that is DSA-negative and C4d-negative (40). These categories are clinically important but remain areas of uncertainty, especially because microvascular inflammation is not specific for AMR. T-cell-mediated rejection, thrombotic microangiopathy, infection-related injury, ischemia-reperfusion injury, recurrent or *de novo* glomerular disease, drug-associated endothelial injury, and surgical or vascular complications should be considered in the differential diagnosis.

### 3.3 Molecular diagnosis and biomarkers

Biopsy-based transcriptomic profiling and molecular diagnostics can reveal signals of endothelial activation, interferon-gamma signaling, NK-cell and macrophage-associated transcripts, and other rejection-related injury patterns (42–45). They are most useful when the biopsy is read in context, alongside histology, DSA, C4d staining, and the clinical course. Their limits are just as important. Access varies, platforms do not perform in exactly the same way, and validation is still incomplete. Noninvasive biomarkers are developing at the same time. Donor-derived cell-free DNA (dd-cfDNA) is a sensitive marker of allograft injury and can help identify patients who need a closer look, but it is not specific for AMR (46–48). It should be interpreted together with DSA, kidney function, proteinuria, infection status, time from transplantation, biopsy findings, and the assay platform used. Single-cell and spatial transcriptomic methods are useful for mechanism work, but they are not ready for routine diagnosis until clearer thresholds and clinical use cases are established.

### 3.4 Non-HLA antibodies and endothelial injury

Non-HLA antibodies are increasingly recognized as potential contributors to endothelial injury and AMR-like phenotypes. Candidate targets include angiotensin II type 1 receptor (AT1R), endothelin receptor type A (ETAR), MICA (MHC class I chain-related protein A), and other endothelial or autoantigen-directed antibodies (49–51). These antibodies may be associated with vascular injury, microvascular inflammation, and graft dysfunction, particularly in cases without detectable HLA-DSA. However, clinical implementation remains limited by assay heterogeneity, uncertain positivity thresholds, variable reproducibility, and incomplete proof of pathogenicity. At present, non-HLA antibody testing should be interpreted as complementary risk assessment rather than a substitute for integrated histological, serological, and clinical evaluation. Evidence supporting phenotype-specific treatment strategies for non-HLA antibody-associated injury remains limited.

## 4 Therapeutic strategies for AMR

Treatment of AMR should be discussed according to phenotype, timing, and certainty of evidence. Widely used regimens such as plasma exchange and IVIG are supported mainly by expert consensus and heterogeneous observational data rather than definitive randomized evidence for long-term graft survival (52). Other interventions have been tested in randomized trials with neutral, negative, or phenotype-specific results, and many emerging therapies are supported only by small cohorts, case reports, or preclinical rationale. For each strategy, the relevant AMR phenotype, study design, endpoints, limitations, toxicities, monitoring requirements, and clinical status should be distinguished.

### 4.1 Management of DSA clearance

For selected patients with active AMR, plasma exchange (PE) combined with intravenous immunoglobulin (IVIG) is widely used as an initial treatment strategy, usually with corticosteroids and optimization of maintenance immunosuppression (52–55). This approach aims to reduce circulating DSA and modulate Fc receptor- and complement-mediated injury. However, protocols differ substantially in the number of PE sessions, IVIG dose, concomitant corticosteroids, and additional agents, and robust randomized evidence showing improved long-term graft survival remains limited. Immunoadsorption can more selectively remove IgG and may reduce some complications of nonselective plasma exchange, but available evidence is mainly derived from nonrandomized cohorts and center-specific experience (56). Its use is also limited by cost, technical demands, and availability.

### 4.2 B-cell depletion therapy

Rituximab removes CD20-positive B cells, which is why it has been tried in AMR, usually together with plasma exchange and IVIG. The clinical signal, however, has not been consistent. Some observational cohorts and single-center reports describe improvement in selected patients, but the multicenter, double-blind randomized RITUX-ERAH trial did not show a clear clinical or histological advantage when rituximab was added to standard treatment for acute AMR (57). This result fits with the biology. Rituximab does not target mature plasma cells or long-lived plasma cells, because these cells do not express CD20. DSA production can therefore continue even after circulating B cells have been depleted (58). For this reason, rituximab should be viewed as a possible add-on treatment in selected AMR settings, not as a treatment that can reliably control AMR on its own.

Anti-CD38 monoclonal antibodies are aimed at a different part of the response. By targeting CD38-expressing plasma cells, and in some settings NK-cell populations, they may address antibody production more directly than rituximab. The evidence for felzartamab and daratumumab is still quite different. Felzartamab has moved into prospective clinical evaluation for late or chronic active AMR, while daratumumab in kidney allograft AMR is still supported mainly by case reports, small series, and pilot experience (59–63). These drugs should therefore be introduced cautiously. Their possible benefit has to be weighed against infection, cytopenias, hypogammaglobulinemia, NK-cell depletion, and the possibility that DSA may persist or rebound after treatment.

### 4.3 Complement inhibition

Complement inhibition is biologically attractive in AMR phenotypes characterized by complement-binding DSA, C4d deposition, or early complement-dependent injury (64). Eculizumab blocks C5 cleavage and can reduce terminal complement activation, but its effect is phenotype-specific and should not be generalized to all severe or chronic active AMR. Studies in sensitized or positive-crossmatch transplantation suggest that terminal complement blockade may reduce early AMR in selected high-risk settings, whereas evidence in established chronic antibody-mediated injury is limited and inconsistent (65–67). Complement-independent mechanisms, including Fc gamma receptor-mediated NK-cell activation and endothelial activation, may persist despite C5 blockade. Treatment also requires careful prevention and monitoring of meningococcal and other encapsulated bacterial infections (68). C1 esterase inhibitor targets proximal complement and contact pathways, but available randomized evidence remains preliminary and insufficient to establish routine use (69).

### 4.4 Anti-inflammatory therapy

The IL-6 pathway is involved in Tfh-cell function, B-cell differentiation, plasma-cell survival, inflammation, and alloimmune injury (70, 71). Tocilizumab and clazakizumab have therefore been investigated in chronic active AMR or refractory microvascular inflammation. Retrospective studies and small clinical experiences suggest possible stabilization of eGFR decline or reduction in inflammatory activity in selected patients, but the evidence remains heterogeneous and patient selection is unresolved (70–73). Ongoing and completed trials should be interpreted with attention to AMR phenotype, baseline chronicity, endpoints, infection risk, cytopenias, liver enzyme abnormalities, gastrointestinal complications, and durability of response. IL-6 pathway inhibition should be presented as an emerging strategy rather than established standard therapy.

### 4.5 Targeted clearance of plasma cells

Proteasome inhibitors target antibody-secreting plasma cells and have been explored as DSA-reducing therapies. Early experimental studies, case series, and small observational reports suggested that bortezomib might reduce DSA or histologic activity in some AMR settings (74, 75). However, the randomized, double-blind, placebo-controlled BORTEJECT trial in late AMR did not show prevention of eGFR decline, reduction in DSA, or improvement in histologic or molecular disease features, and treatment was associated with gastrointestinal and hematologic toxicity (76). Conclusions regarding bortezomib should therefore be substantially moderated, especially for late or chronic active AMR. Carfilzomib has greater pharmacological selectivity and may have a different toxicity profile, but there is no robust comparative clinical evidence showing superiority over bortezomib in kidney transplant AMR. It should be described as investigational, with evidence limited to small and predominantly noncomparative studies (77).

### 4.6 Optimization of immunomodulation and maintenance therapy

Optimization of maintenance immunosuppression is central to preventing *de novo* DSA and late AMR. Tacrolimus exposure, intra-patient variability, underimmunosuppression, medication nonadherence, and mycophenolate dose reduction should be considered when evaluating patients with *de novo* DSA or late AMR (3, 78, 79). Belatacept blocks CD28-CD80/86 costimulation and may reduce *de novo* DSA formation in selected clinical contexts, but this potential should be balanced against the risk of early cellular rejection and the lack of evidence supporting routine use as direct treatment for established AMR (80, 81). *Post hoc* analyses of the BENEFIT and BENEFIT-EXT trials showed a lower incidence of *de novo* DSA with belatacept than with cyclosporine, and long-term observational data support its use mainly as a maintenance or conversion strategy rather than a direct treatment for established AMR (82–84). Experimental studies combining belatacept with additional immunomodulatory approaches, such as BTLA overexpression, should not be used to support broad clinical prophylaxis recommendations. mTOR inhibitors may influence B-cell and Tfh biology, but their role must be weighed against proteinuria, wound healing, rejection risk after CNI minimization, and patient-specific immunologic risk (78, 85) (**Table 1**).

**Table 1:** Evidence-based summary of pharmacological and translational strategies for AMR after kidney transplantation.

Therapy	Main target or mechanism	Relevant AMR phenotype or clinical setting	Evidence type	Key limitations and monitoring	Clinical status	Main clinical endpoints evaluated	Key references
Plasma exchange plus IVIG	Rapid reduction of circulating DSA; Fc receptor and complement modulation	Selected active AMR, usually with corticosteroids and maintenance immunosuppression optimization	Expert consensus; heterogeneous observational data; limited randomized evidence for long-term graft survival	Protocol heterogeneity; coagulation factor and protective antibody removal; infection and volume risks	Widely used initial therapy, not supported by high-certainty evidence for all phenotypes	DSA reduction; graft function; histologic AMR activity; graft survival; infection and treatment-related complications	(52, 54, 55)
Immunoadsorption	Selective IgG removal	Selected active AMR or high DSA burden in center-specific protocols	Mostly retrospective cohorts and center experience	Cost, technical demands, availability, and limited comparative outcome data	Off-label or center-specific adjunct	IgG/DSA reduction; short-term graft function; AMR response; treatment complications	(56)
Rituximab	CD20-positive B-cell depletion	Adjunctive therapy in selected active AMR; limited effect on mature plasma cells	Mixed observational evidence; RITUX-ERAH randomized placebo-controlled trial was neutral	Does not target CD20-negative plasma cells; infection and hypogammaglobulinemia monitoring	Adjunctive off-label therapy with uncertain efficacy	Graft function; histologic response; DSA change; graft survival; infection and hypogammaglobulinemia	(57, 58)
Bortezomib	Proteasome inhibition and plasma-cell apoptosis	Explored in DSA-positive late AMR and selected refractory cases	BORTEJECT randomized placebo-controlled trial negative for late AMR	No clear benefit for eGFR, DSA, histology, or molecular endpoints in late AMR; gastrointestinal and hematologic toxicity	Not established for late or chronic active AMR	eGFR decline; DSA levels; histologic activity; molecular rejection features; gastrointestinal and hematologic toxicity	(74–76)
Carfilzomib	Second-generation proteasome inhibition	Investigational plasma-cell targeting strategy	Pediatric, small-scale, and predominantly noncomparative clinical experience	No robust comparative evidence versus bortezomib in kidney AMR; toxicity profile requires monitoring	Investigational	DSA reduction; graft function; AMR response; treatment-related toxicity	(77)
Eculizumab	C5 blockade and terminal complement inhibition	Complement-dependent injury, positive crossmatch or selected high-risk settings	Prospective and pilot randomized data; phenotype-specific results	Limited effect on complement-independent injury; meningococcal and encapsulated bacterial infection risk	Selected off-label use, not generalizable to all AMR	Early AMR incidence; graft function; complement-mediated injury; graft survival; meningococcal or encapsulated bacterial infection	(64–68)
Tocilizumab	IL-6 receptor blockade	Chronic active AMR, refractory microvascular inflammation, or selected inflammatory phenotypes	Literature review; retrospective cohorts; center experience	Unresolved patient selection, durability, infection risk, cytopenias, liver enzyme and gastrointestinal monitoring	Emerging strategy, not established standard therapy	eGFR slope; microvascular inflammation; DSA changes; histologic activity; infection; cytopenias; liver enzyme abnormalities; gastrointestinal events	(71, 72)
Clazakizumab	IL-6 neutralization	Chronic active AMR or selected inflammatory AMR phenotypes	Early clinical studies and trial protocol/prospective evaluation	Unresolved patient selection and durability; infection, cytopenias, liver enzyme abnormalities, and gastrointestinal monitoring	Emerging strategy, not established standard therapy	eGFR slope; DSA changes; biopsy-based activity; microvascular inflammation; infection; cytopenias; liver enzyme abnormalities; gastrointestinal events	(70)
Felzartamab	CD38-positive plasma-cell and immune-cell targeting	Late or chronic active AMR; refractory DSA-producing phenotypes	Prospective clinical evaluation and drug evaluation	Infection, cytopenias, hypogammaglobulinemia, NK-cell depletion, and rebound or persistent DSA require monitoring	Investigational or early clinical strategy	DSA reduction or persistence; biopsy activity; graft function; plasma-cell depletion; infection; cytopenias; hypogammaglobulinemia; NK-cell depletion	(59, 60)
Daratumumab	CD38-positive plasma-cell and immune-cell targeting	Refractory or late AMR with persistent DSA	Case report, small series, and pilot experience	Infection, cytopenias, hypogammaglobulinemia, and rebound or persistent DSA require monitoring	Investigational or early clinical strategy	DSA reduction or persistence; graft function; biopsy activity; plasma-cell depletion; infection; cytopenias; hypogammaglobulinemia	(59, 61–63)
Belatacept	CD28-CD80/86 costimulation blockade	Maintenance or conversion context; potential reduction of *de novo* DSA in selected patients	Clinical transplant experience and mechanistic rationale; not direct established AMR treatment	Early cellular rejection risk; limited evidence for established AMR treatment	Maintenance strategy in selected contexts, not routine AMR therapy	*de novo* DSA formation; rejection incidence; graft function; graft survival; conversion outcomes	(82–84)

AMR, antibody-mediated rejection; DSA, donor-specific antibody; IVIG, intravenous immunoglobulin; MVI, microvascular inflammation; C5, complement component 5; NK cell, natural killer cell.

## 5 Future research directions of AMR

### 5.1 Precision immune typing and individualized treatment

Two prerequisites exist to overcome AMR: precise immune typing and individualized treatment. High-throughput technologies like single-cell omics and spatial transcriptomics allow us to gain a comprehensive understanding of the local immune microenvironment during AMR with unprecedented resolution (86, 87). Systematic analysis of transcriptome data from renal transplant biopsy tissues identifies specific molecular signatures for acute and chronic active AMR, revealing differences in their pathogenesis and potential therapeutic targets (87, 88). Classifying patients from a molecular perspective allows distinguishing AMR subtypes at the clinical level, which lays the foundation for differentiating targeted treatments. Analysis of genetic polymorphisms also holds promise for predicting treatment responses. Genetic variations of HLA-G between donors and recipients are closely related to the prognosis of renal transplant rejection, particularly regarding the immunoregulatory role of the HLA-G/LILRB1 ligand-receptor axis (89). Specifically, the polymorphism in the 3’UTR region of the donor’s HLA-G and variation in the promoter of the recipient’s LILRB1 are respectively associated with T-cell-mediated rejection and the risk of AMR (90); other variations such as Fcγ receptors or complement-related genes will impact the treatment response of patients to certain drugs (such as anti-CD20 monoclonal antibodies and complement inhibitors). Artificial intelligence algorithms have begun to play a role in early warning and preventive intervention of AMR (91). Integrating clinical data, serological indicators, and pathology results into one analytical framework, we can build an AMR risk model with high predictive accuracy (91) so that we can identify high-risk individuals before the onset of AMR. Clinicians may then change the immunosuppressive regimen or increase monitoring frequency according to this information to delay or prevent disease occurrence.

### 5.2 New biological agents, cell therapies, and gene editing

Future strategies should be clearly separated from therapies currently supported by clinical evidence in kidney transplant AMR. CD3-CD20 bispecific antibodies are established in hematologic malignancies and provide a conceptual rationale for dual immune-cell targeting, but current lymphoma data should not be presented as direct evidence for AMR treatment (92). CAR-T, chimeric HLA antibody receptor T cells, and CAR-Treg approaches remain preclinical or early translational concepts in transplantation (93, 94). Similarly, regulatory T-cell therapy in kidney transplantation has mainly focused on safety, feasibility, tolerance induction, or immunosuppression minimization rather than established treatment of AMR (95–99). Gene editing of donor HLA may reduce alloantigenicity in experimental systems, but major barriers include NK-cell missing-self responses, non-HLA immunogenicity, off-target effects, organ engineering logistics, ethical issues, and regulatory requirements (49, 100).

### 5.3 Multicenter clinical trials and translational research

A persistent limitation in AMR research is the scarcity of adequately powered, phenotype-specific randomized trials. Future trials should define AMR phenotypes according to Banff criteria and report the factors that shape treatment response and outcome interpretation, including baseline chronic injury, DSA features, biopsy findings, kidney function, proteinuria, patient and graft survival, infection events, and patient selection criteria (52). dd-cfDNA may help detect graft injury and guide the decision to perform a biopsy, but it should not be treated as an AMR-specific surrogate endpoint on its own. Its interpretation should be combined with DSA, histology, molecular findings, renal function, proteinuria, and infection assessment (46, 101). Multicenter biobanks linking serum, urine, biopsy tissue, transcriptomic data, and long-term follow-up will be useful for developing interventions that are biologically grounded and feasible to test in clinical studies.

## 6 Conclusion

AMR is better viewed as a group of related injury patterns than as a single treatment target. In individual patients, DSA, non-HLA antibodies, complement activation, Fc receptor-mediated cellular injury, endothelial activation, and chronic microvascular remodeling may contribute to different extents. Banff updates, biopsy-based transcriptomics, DSA profiling, and noninvasive biomarkers have made AMR easier to recognize, but none of them can stand alone. They still have to be read together with histology, the clinical course, and graft function. Treatment remains difficult because the evidence base is uneven, responses differ across phenotypes, and randomized trial data are still limited. What comes next is more precise AMR classification, trials in better defined patient groups, and treatment choices that follow the phenotype, the degree of chronic injury, the dominant immune mechanism, and the safety profile.
